# Whole genome sequencing reveals significant intra-hospital clonal transmission and a potential multidrug resistant and hypervirulent sequence cluster of *Corynebacterium striatum*

**DOI:** 10.1080/22221751.2025.2563795

**Published:** 2025-10-14

**Authors:** Menglan Zhou, Jiawei Chen, Tingting Zhang, Lingli Liu, Jingjia Zhang, Wei Kang, Hongtao Dou, Dingding Li, Lina Guo, Ying Zhao, Yali Liu, Renyuan Zhu, Hongli Sun, Zhengyin Liu

**Affiliations:** a Department of Laboratory Medicine, Peking Union Medical College Hospital, Chinese Academy of Medical Sciences and Peking Union Medical College, Beijing, People’s Republic of China; b Laboratory Medicine Center, Department of Clinical Laboratory, Zhejiang Provincial People's Hospital, Affiliated People's Hospital, Hangzhou Medical College, Hangzhou, People’s Republic of China; c Beijing Chest Hospital, Capital Medical University, Beijing Tuberculosis and Thoracic Tumor Institute, Beijing, People’s Republic of China; d Department of Infectious Disease, Peking Union Medical College Hospital, Chinese Academy of Medical Sciences and Peking Union Medical College, Beijing, People’s Republic of China

**Keywords:** *Corynebacteritum straitum*, hypervirulent, multidrug resistant, molecular epidemiology

## Abstract

With increasing reports about *Corynebacteritum straitum*, its role as an emerging human pathogen is been recognized. However, few studies have explored the genomic epidemiology of *C*. *straitum* in China. A total of 263 isolates collected in various specimens from 2021 to 2022 were analyzed from a tertiary hospital in China. Nearly all isolates (98.5%, 259/263) were multidrug resistant (MDR). The highest resistance was observed for clindamycin (96.5%), followed by ciprofloxacin (95.0%) and erythromycin (93.5%). Genome sequencing indicated a significant prevalence of intra-hospital clonal transmission, involving six distinct clones (clone one-six). An average of 7.35 antimicrobial resistance genes and 10.65 virulence genes were identified in each strain. Genomic analysis identified a potentially hypervirulent sequence cluster (designated SC3) exhibiting near-universal carriage of four pilus assembly genes (*spaH*, *spaI*, *srtD*, and *srtE*), whereas these genes occurred at significantly lower frequencies in other sequence clusters. Further analysis estimated the emergence of SC3 strains around 1929.56 (95% HPD: 1863.41-1995.71). Phenotypic virulence assays demonstrated that *C. straitum* strains carrying the *spaH*, *spaI*, *srtD*, and *srtE* genes exhibited significantly higher virulence compared to strains lacking these genes. Our results revealed that multiple MDR *C*. *straitum* colones were circulating in the hospital. A suggestive MDR and hypervirulent SC3 was identified with potential evolutionary advantage and enhanced transmission capability.

## Introduction

*Corynebacterium striatum* is a Gram-positive bacterium, belonging to class Actinobacteria, order Actinomycetales, and family Corynebacteriaceae. It is commonly seen in natural environments, especially in soil and water, also constituting as normal flora of human skin and nasal mucosa [1–4]. In the past, *C. striatum* has been considered a contaminant of clinical specimens in most circumstances. However, in recent decades, various infections including respiratory, wound, bacteremia, endocarditis, urinary tract infections and even meningitis due to *C. striatum* have been reported [5–9]. Moreover, as an emerging human pathogen, *C. striatum* has been associated with nosocomial outbreaks in several countries [10–16]. Apart from the increasing infection incidents, *C. striatum* also developed resistance against multiple antimicrobials, including β-lactams, aminoglycosides, tetracyclines, macrolides, and fluoroquinolones. Reported proportion of multidrug resistant (MDR) *C. striatum* ranged from 77.6% in Korea to higher than 95% in China and the US [17–19]. The outbreak potential and the MDR pattern further complicates the treatment of *C. striatum* infections. Thus, it’s of great significance to timely recognize the nosocomial transmission and unveil the resistance mechanism of *C. striatum*.

Whole-genome sequencing (WGS) combined with bioinformatics analysis serves as a powerful tool to investigate potential epidemics and track nosocomial transmission. Genomic analysis also reveals detailed genetic determinants mediating antibiotic resistance and pathogenicity. A previous study suggested possible nosocomial epidemic of MDR *C. striatum* in three regions of China through WGS analysis with *ermX* being the most predominant resistance gene [18]. Nevertheless, being a burgeoning pathogen, large-scale genomic studies exploring the genetic characteristics of *C. striatum* were relatively few.

In this study, we retrospectively analyzed the susceptibility pattern and genomic characteristics of *C. striatum* isolates in a tertiary hospital in China. Pan-genomic analysis of the *C. striatum* species, including 263 newly sequenced clinical isolates in our research and 355 currently published genomes retrieved from public databases was performed, focusing on the genomic diversity and evolution of *C. striatum* isolates.

## Materials and methods

### Strain identification and antimicrobial susceptibility testing

We performed a retrospective study of 263 *C. striatum* isolates recovered from a variety of clinical specimens in Peking Union Medical College Hospital (PUMCH), from 2021 to 2022. For each patient, only the first isolate during the entire collection period is acceptable unless the isolation interval of multiple *C. striatum* isolates was more than one week and genome analysis assigned them to different clones. For each sample included, we have carefully reviewed the clinical and microbiological information. The inclusion criteria as follows: (1) microscopy suggesting gram-positive bacilli dominating or neutrophil phagocytosis; (2) culture revealing *C. striatum* as the only or the dominating pathogen; (3) the administration of anti-gram-positive bacteria drugs after the isolation of *C. Striatum*. Samples met at least one of the three criteria were included.

All the isolates were initially identified using Vitek matrix-assisted laser desorption ionization-time of flight mass spectrometry (MALDI-TOF MS) (bioMérieux, France) and then submitted for whole-genome sequencing. The minimum inhibition concentrations (MICs) of 18 antimicrobial agents, namely, penicillin, cefotaxime, ceftriaxone, cefepime, meropenem, tetracycline, doxycycline, ciprofloxacin, clindamycin, erythromycin, gentamicin, rifampicin, vancomycin, linezolid, daptomycin, chloromycetin, teicoplanin and tigecycline were determined using broth microdilution method according to CLSI guidelines. *Escherichia coli* ATCC 25922 and *Streptococcus pneumoniae* ATCC 49619 were used as quality controls. MICs were interpreted using CLSI interpretative breakpoints (CLSI document M45, the 3rd edition) [20]

### Whole-genome sequencing and analysis

The genomic DNA extracted from all 263 *C. striatum* isolated from this study was subjected to draft-genome sequencing on illumina novaseq 6000 system and five isolates were further sequenced using PacBio RSII sequencer. Following the removal of low-quality sequences and adapters, the reads were de novo assembled using the SPAdes Genome Assembler (v3.11.1) [21] and hybrid assembled using Unicycler (v0.4.6) [22]. To enrich the analyzed data, a total of 355 genomes of *C. striatum* were retrieved from the NCBI genome database. QUAST (v5.2.0) was used to assess the quality of all *C. striatum* genome [23], and retaining those meeting quality thresholds of N50 ≥ 6,000 bp and N90 ≥ 2,000 bp. The pairwise average nucleotide identity (ANI) values of *C. striatum* genome sequences were calculated using pyANI. (v0.2.12) [24]. Antimicrobial resistance genes (ARGs) and virulence genes (VGs) were characterized with abricate (v1.0.1) (Seemann T. Abricate. Github. Available from: https://github.com/tseemann/abricate) screened against the ResFinder database [25] and Virulence Factors of Pathogenic Bacteria Database (VFDB), respectively, with a threshold of 70% coverage and identity [26]. Easyfig (v2.2.2) was used to visualize the linear alignment of the genetic environment surrounding most common ARGs and four VGs (*spaH*, *srtD*, *srtE*, and *spaI*) [27]. The set of mobile genetic elements (MGEs) including genomic islands (GIs), integrative conjugative elements (ICEs), integron, prophage, transposes (Tns), and insertion sequences (ISs) in all *C. striatum* isolates were analyzed using Mobilome Prediction at VRprofile2 database [28].

### Timed phylogeny reconstruction, transmission dynamics and phylogenetic analysis

Phylogenetic analysis utilized an assembly-based core-genome single nucleotide polymorphisms (SNPs) alignment constructed with parsnp (v1.7.4) from the Harvest suite, incorporating recombination detection [29]. A maximum-likelihood phylogenetic tree, based on the recombination-free core-genome SNPs, was generated using RAxML (v0.6.0) with the GTR + Gamma model and 100 bootstrap replicates [30]. iTOL (v3.0) was employed for annotating the tree, integrating the background information and molecular characteristics of the strains [31]. The sequence clusters (SCs) within the tree were identified through a fast hierarchical Bayesian analysis (fastbaps) model, with the “optimised.symmetric” type selected and other parameters kept at their default settings [32]. To investigate clonal transmission within the hospital, we defined clones as groups with fewer than 20 SNPs and consisting of more than 10 strains, based on the recent studies of *C. striatum* intra-hospital transmission [33]. The analysis of intra- and inter-ward transmission of the same clone was conducted using regentrans package in R (v4.1.1) [34]. To estimate the origin time of the analyzed strains, we ran BactDating package in R (v4.1.1) with mixed-gamma evolutionary models for 10,000,000 MCMC steps [35]. Additionally, the transmission route of clone one was inferred using TransPhylo package in R (v4.1.1) with 10,000,000 MCMC steps [36]. The effective sample size (ESS) values for all parameters were greater than 200, indicating adequate sampling of the posterior distribution.

### Comparative genomic analysis

The assemblies of *C. striatum* were annotated with Prokka (v1.14.6) [37], and the pan-genome was calculated using Roary (v3.13.0) [38]. Scoary (v1.6.16) [39] was employed for comparative genomic analysis of *C. striatum* between the SC3 strains, which has the most protein-coding genes (CDS), and the other strains or other sequence cluster strains (SC1, SC17, SC2, SC21, and SC7) within the same clade as SC3, achieving both sensitivity and specificity of ≥80% and *P* < 0.01 (Bonferroni test). The identified differential genes were annotated and analyzed using eggNOG-mapper (v2) [40]. The annotations were further analyzed for Gene Ontology (GO) and Kyoto Encyclopedia of Genes and Genomes (KEGG) pathway enrichment analysis using clusterProfiler package in R [41].

### Virulence assessed by cell viability assay

Cell cytotoxicity was assessed by measuring the lactate dehydrogenase (LDH) released from damaged cells, as described in previous studies [42]. Briefly, human alveolar epithelial A549 cells were seeded in 96-well plates at a density of 1 × 10⁴ cells/well and incubated for 24 hours. After discarding the culture medium, cells were treated with 100 µL of bacterial suspension (1 × 10⁸ CFU/mL) per well for an additional 6 hours. The 50 µL supernatant from each well was then transferred to a new plate, to which 50 µL of working solution was added. The mixture was incubated in the dark at 37°C for 30 minutes, followed by the addition of 50 µL of stop solution. The cell-free culture medium served as the blank control, while the medium-treated wells containing seeded cells served as the negative control. Cells lysed with LDH-releasing reagent were used as the positive control, and cell-free medium with LDH-releasing reagent served as the high-background control. Cytotoxicity was calculated using the following formula: (OD_490_ of sample-OD_490_ of negative control)/[(OD_490_ of positive control−OD_490_ of high-background control)−(OD_490_ of negative control−OD_490_ of blank control)] × 100%. All experiments were performed in triplicate.

### Adhesion assay using cell models

As previously described [42], A549 cells were cultured in 12-well plates at a density of 1 × 10⁵ cells per well and exposed to bacteria at a multiplicity of infection (MOI) of 100:1 for 3 hours. Afterward, non-adherent bacteria were removed by washing the cells with PBS. To quantify the number of bacteria that had adhered to the cells, the cells were lysed with 1 mL of 0.1% saponin for 20 minutes followed by plating onto MH agar plates for bacterial counting. All experiments were performed in triplicate.

### Virulence testing in the *Galleria mellonella* infection model

The virulence of the strain was also evaluated using the *Galleria mellonella* infection model, as previously described [42]. Briefly, 250–300 mg wax moth larvae were injected with 10μL of bacterial suspension (1 × 10⁵ CFU/mL) and incubated at 37°C for 72 hours. Larval survival was recorded at 24, 48, and 72 hours post-infection. Normal saline-injected larvae served as the negative control. Each strain was tested in ten larvae. All experiments were performed in triplicate.

## Results

### Epidemiological distribution of the isolates

By application of the criteria above, a total of 263 *C. striatum* isolates were obtained from 255 patients admitted to PUMCH during 2021-2022. Due to the limited medical record, the medication and clinical outcomes of outpatients were not traceable. Of these, 143 out of 197 inpatients were administrated anti-gram-positive bacteria drugs after isolation of *C. striatum*; 217 samples isolated *C. Striatum* only; 37 samples isolated *C. Striatum* and other bacteria; 9 samples isolated *C. Striatum* and other *Candida* spp.; the latter two categories were included under the circumstances that *C. Striatum* was the dominating pathogen as suggested by microscopy or culture. Among these, 67.8% were isolated from male patients and 32.2% were from female patients. The mean age of the patients was 65.8 ± 17.2 years with the majority of the strains isolated from patients ≥65 years old. More than 90% of the patients were suffered from different morbidities, the majority of which were chronic disorders including hypertension, diabetes mellitus, chronic obstructive pulmonary disease, etc. Patients with immunocompromised status accounted for 30.6%, with solid tumor dominating (Table 1). The calculated the 30-day mortality was 13% (25/197) for inpatients.

**Table 1.** Clinical information of 263 *C. striatum* isolates.

Variables	Number	Percent (%)
**Sex**		
Male	173	67.8
Female	82	32.2
Total	255	100.0
**Age groups**		
19–35	21	8.2
36–50	22	8.6
51–64	50	19.6
≥65	162	63.5
Total	255	100.0
**Comorbidities**	**Number**	**Percent**
Hypertension	30	11.8
Diabetes mellitus	19	7.5
Neurological disorders	12	4.7
Coronary heart disease	4	1.6
Chronic obstructive pulmonary disease	4	1.6
Two of the above	36	14.1
Three of the above	25	9.8
Others	21	8.2
None	26	10.2
**Immunocompromised status**	** **	
Solid tumor	44	17.3
Systemic autoimmune disease	19	7.5
Hematology Malignancy	15	5.9
Total	255	100.0
**Department**	** **	
Emergency	81	31.8
Intensive care unit	64	25.1
Respiratory	20	7.8
Neurosurgery	18	7.1
Neurology	12	4.7
Hematology	9	3.5
Infectious diseases	4	1.6
Gerontology	4	1.6
Orthopedics	3	1.2
Gastroenterology	2	0.8
Immunology	2	0.8
General internal medicine	2	0.8
Dermatology	2	0.8
Oncology	2	0.8
Other wards	15	5.9
Outpatient	15	5.9
Total	255	100.0
**Specimen**		
Sputum	174	66.2
Tracheal aspiration	52	19.8
Bronchoalveolar lavage fluid	10	3.8
Wound secretions	6	2.3
Urine	5	1.9
Blood	4	1.5
Pus	2	0.8
Ascites	2	0.8
Others	8	3.0
Total	263	100.0

Most of the strains were isolated from patients in the emergency department, followed by intensive care unit, respiratory and neurosurgery, each accounting for 31.8%, 25.1%, 7.8% and 7.1%. *C. striatum* strains were isolated from various types of specimens. Except for 6 wound secretions, 5 urines, 4 blood, 2 each of pus and ascites, nearly 90% were from respiratory samples (Table 1). Furthermore, we retrieved an additional 355 strains from the NCBI database as supplementary data for our analysis. These strains span a wide isolation period from 1900 to 2022, with the highest proportions isolated in 2017 (30.7%), 2018 (23.4%), and 2016 (15.5%). The strains originated from 12 different countries. China was the predominant source country (74.4%), followed by the United States (8.2%) and Australia (5.9%). Regarding specimen types, sputum was the most common source for the NCBI strains (63.7%), with other sources including blood (5.1%), swabs (3.4%), and tissue (2.5%).

### Antimicrobial susceptibility profiles

The highest resistance rate was observed for clindamycin (96.5%), followed by ciprofloxacin (95.0%), erythromycin (93.5%), cefotaxime (93.4%), penicillin (93.1%), ceftriaxone (92.3%) and cefepime (91.2%). All the isolates were susceptible against linezolid, vancomycin and daptomycin. There were no breakpoints for teicoplanin and tigecycline but the MIC_50_ and MIC_90_ values were at low levels (Table 2). MDR, defined as nonsusceptibility to at least one agent in three or more antimicrobial categories, was observed in 98.5% (259/263) of isolates.

**Table 2.** In vitro susceptibility of 263 *C. striatum* isolates against 18 antimicrobial drugs.

Antibiotics	S (%)	I (%)	R (%)	MIC_50_	MIC_90_	MIC range (μg/mL)
Penicillin	0	6.9	93.1	>32	>32	<0.12–>32
Cefotaxime	5.4	1.2	93.4	>32	>32	<0.25–>32
Ceftriaxone	3.1	4.6	92.3	>32	>32	0.5–>32
Cefepime	8.8	0	91.2	>32	>32	<0.25–>32
Meropenem	12.3	0.8	86.9	>32	>32	<0.12–>32
Tetracycline	32.7	2.3	65.0	16	32	<0.06–>32
Doxycycline	72.2	27.8	0	4	8	<0.06–8
Ciprofloxacin	5.0	0	95.0	>32	>32	<0.06–>32
Clindamycin	0.8	2.7	96.5	32	>32	0.25–>32
Erythromycin	5.0	1.5	93.5	32	>32	<0.12–>32
Gentamicin	71.2	6.5	22.3	4	>64	<1–>64
Rifampicin	92.7	0	7.3	<0.25	<0.25	0.25–>32
Vancomycin	100	NA	NA	<0.25	<0.25	<0.25–0.5
Linezolid	100	NA	NA	0.25	0.25	<0.12–0.5
Daptomycin	99.6	NA	NA	<0.064	<0.064	<0.064–16
Chloromycetin	NA	NA	NA	2	32	0.25–>32
Teicoplanin	NA	NA	NA	<0.25	<0.25	<0.25
Tigecycline	NA	NA	NA	<0.015	<0.015	<0.015–0.5

### Assembly quality control and ANI values assay of all genomes

We have rigorously evaluated the assembly quality metrics for all genomes in this study including both de novo assembled isolates and publicly available genomes downloaded from NCBI using QUAST (Table S1). The assembly quality of strains collected in this study was generally high, with contig numbers ranging from 20 to 219 and 79.1% of strains containing fewer than 100 contigs. Their N50 and N90 values ranged from 26,390 to 402,216 and 6,840 to 189,133, respectively. For strains downloaded from NCBI, assembly quality was similarly high, with 68.7% containing fewer than 100 contigs; N50 and N90 values ranged from 6,058 to 3,031,488 and 2,259 to 3,031,488, respectively. To ensure that all strain genomes belong to *C. striatum*, we calculated the pairwise ANI values by comparing the strains collected in this study and those downloaded from NCBI with the *C. striatum* reference genome (GCF_016728105.1). The results showed that the ANI value between the strains in this study and the reference sequence was ≥97.67%, while the ANI value for the strains downloaded from NCBI was ≥97.62% (Figure S1). Based on the species classification threshold of ≥95%, both the strains from this study and those downloaded from NCBI belong to *C. striatum*.

### Clonal transmission of *C. striatum* isolated from this study

**Figure 1.**
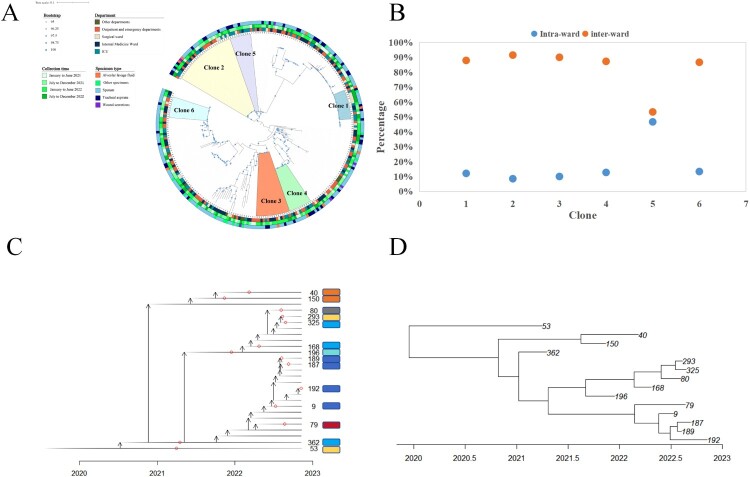
Phylogenetic and clonal transmission analysis of 263 *C. striatum* isolated from this study. A. The phylogenetic tree of *C. striatum* isolates and corresponding background information. The numbers and colors on the phylogenetic tree represent different clones of *C. striatum*. B. Analysis of transmission routes intra- and inter-ward for the same clone of *C. striatum*. C. The transmission tree of clone one of *C. striatum*. The different colors on the right represent different departments. D. Timed phylogeny reconstruction of clone one of *C. striatum*.

To elucidate the potential correlation between the elevated isolation rate of *C. striatum* and clonal transmission events within healthcare settings, we conducted a phylogenetic analysis of 263 *C. striatum* strains collected in this study. The results showed that strains isolated from the same specimen or department did not cluster together on the phylogenetic tree. However, significant intra-hospital clonal transmission was observed, involving six distinct clones (labeled 1-6) comprising 91 isolates (34.60%) (Figure 1A). Integrated analysis with clinical backgrounds revealed continuous detection of all clones throughout 2021–2022 across multiple departments and specimen types. From these, clone one (subsequently identified as a hypervirulent lineage) was selected for in-depth transmission analysis. This clone disseminated not only within single wards but also across multiple departments (Figure 1B). Time-scaled phylogenetic estimation further indicated its emergence around 2019.20 (95% HPD: 2017.48–2020.94) (Figure 1C, D, S2).

### Antimicrobial resistance genes, virulence genes and mobile genetic elements characteristics of *C. striatum*

The analysis of the ARGs profile of 618 *C. striatum* isolates (263 from this study and 355 from NCBI genome data sets) revealed 21 kinds of ARGs conferring resistance to 9 different classes of antimicrobial agents and the ARG count per isolate varied from 0 to 20, averaging 7.35 genes per isolate (Table S2). Only three strains carried none of the ARGs and nearly 74.1% (194/263) of the strains carried three or more resistance genes at the same time. The prevalence of aminoglycosides resistance genes in *C. striatum* was the highest, whereas β-lactams, trimethoprim, fosfomycin, and fusidic acid resistance genes showed minimal prevalence. When comparing the prevalence of individual genes, it is found that *C. striatum* carried *erm*(X), *aac(3)-XI*, *tet(W)*, and *sul1* at high rates, all exceeding 50%, with values of 89.32%, 77.02%, 63.27%, and 52.27%, respectively. In contrast, the carriage of *aac(6’)-IIa*, *aac(6’)-aph(2'’)*, *bla*_TEM-1A_, *dfrA13*, *erm*(C), *fosD*, *fusB*, *tet*(33), and *tet*(Z) in *C. striatum* was only 0.16%. In addition, point mutations in the strains also contribute to resistance to antimicrobial agents. Mutations in the quinolone resistance-determining region of the *gyrA* gene, with mutation rates of 97.72% for Ser87Phe and 94.30% for Asp91Ala, and a combined mutation rate of 94.30%, were responsible for this resistance. In parallel, a detailed screening in *C. striatum* identified 18 VGs belonging to three classes: iron uptake, adherence, and regulation, with an average of 10.65 genes carried per strain (Table S2). Five VGs, *hmuU*, *irp6A*, *irp6B*, *hmuT* and *fagC*, were present in nearly all *C. striatum* strains, whereas *spaI*, *srtD*, *srtE*, *dtxR*, and *spaH* were only present in about 9% of the strains (Table S4).

The analysis identified diverse MGEs in *C. striatum*, averaging 15.14 per strain. *C. striatum* possesses a high load of genomic islands (averaging 6.67 per strain) and insertion sequences and transposons (averaging 6.16 per strain) (Table S2). The large number of MGEs carried by *C. striatum* mediates the horizontal transfer of its ARGs and VGs. We performed a detailed analysis of the gene environments associated with the most common ARGs carried by *C. striatum* (Figure S1), and the results showed that *erm*(X) and *tet*(W) were often found together, with *IS*Cx1 upstream and *IS*1249 downstream. When *erm*(X) was found alone, it was frequently flanked by *IS*1249. Similarly, aminoglycoside resistance genes, such as *aac(3)-XI*, were flanked by *IS*Cre1 and *IS*Ser1, while *aac(6’)-Ia* and *ant(3'’)-Ia* were associated with *IS*6100. Additionally, *sul1* and *qacE* often form resistance gene clusters with *aac(6’)-Ia* and *ant(3'’)-Ia*, with *IS*6100 present upstream and downstream. Notably, *aph(6)-Id*, *aph(3'’)-Ib*, *aph(3’)-Ia*, and *cmx* form resistance gene clusters, with MGEs such as *IS*Aar26, *IS*26, *IS*5564, and Tn3 found upstream and downstream, and *erm*(X) with *IS*Ser1 downstream. This horizontal transfer could lead to *C. striatum* acquiring resistance to multiple antimicrobial agents. Similarly, MGEs can mediate the horizontal transfer of virulence genes, as demonstrated in subsequent results.

### Genomic and phylogenetic analysis

**Figure 2.**
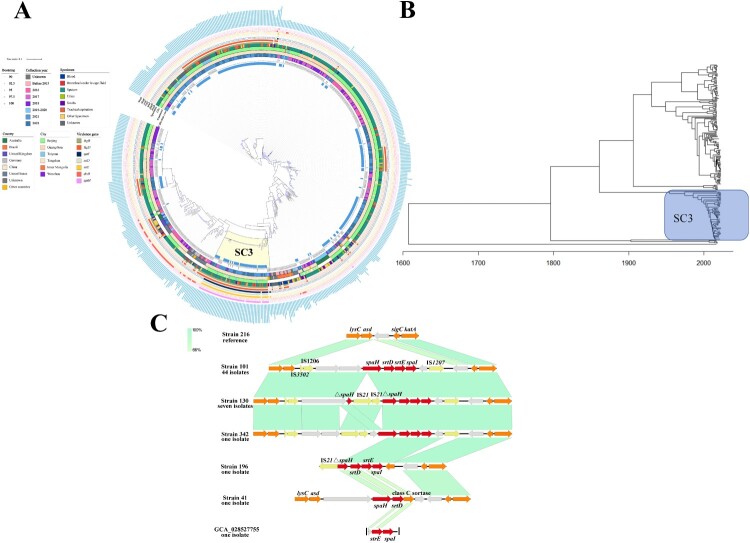
A. Phylogenetic analysis of 618 *C. striatum* isolates and corresponding background information. Given the high number of virulence genes, only those with low prevalence are presented, including *fagB*, *fagD*, *spal*, *srtD*, *srtE*, *dtxR*, and *spaH*. B. The genetic environments surrounding the *spaH*, *spaI*, *srtD*, and *srtE* gene in six representative isolates of *C. striatum.* The red arrows represent the *spaH*, *spaI*, *srtD*, and *srtE* genes, the yellow arrows indicate insertion sequences, and the orange arrows point to functional genes. The grey arrows represent hypothetical proteins. The slashes on GCA_028527755 represented genome fragmentation.

A maximum-likelihood phylogeny was constructed based on 49,657 filtered cgSNPs identified from 618 *C. striatum* strains for further phylogenetic analysis (Figure 2A). The fastbaps population structure analysis clustered the 618 genomes into 21 distinct SCs, designated as SC1 through SC21 (Table S2). We conducted a detailed analysis of the phylogenetic structure (using SCs) and examined the correlations with isolation year, geographical region, specimen type, and isolation source. The results showed that no significant association was observed between strain sequence clusters and isolation year or specimen type (*P* > 0.05). However, significant geographic variation emerged: Australian strains predominantly clustered in SC16 and SC17 (94.7%), while Brazilian strains concentrated in SC6 (87.3%), a clade not detected in Chinese isolates (*P* < 0.001). Strains from the United States and the United Kingdom were predominantly SC21, a type found rarely in China, with only four strains. China had nearly all cluster types, except SC6, suggesting the potential for international transmission of the strains. There were also some differences in cluster types between different cities, with strains from Guangzhou mainly belonging to SC14. The origin of the strains (from this study or those downloaded from NCBI database) showed a strong correlation with their respective clusters. In the strains collected in this study, SC1, SC3, SC4, SC5, SC11, SC12, and SC15 were more common, whereas SC2, SC6, SC7, SC10, SC13, SC14, SC16, SC17, and SC21 were more prevalent in the strains downloaded from the NCBI database (Figure 2A and Table S2). This discrepancy was primarily due to the large geographical differences between the strains collected in this study and those obtained from NCBI database.

We evaluated the abundance of ARGs, VGs, MGEs and CDS within each SC of *C. striatum*, combining with molecular characteristics of strains. Notably, SC3 strains exhibited the highest average counts of VGs, MGEs, and CDS compared with non-SC3 strains (*P* < 0.0001, Table S5). In terms of ARGs, no significant differences were observed but SC3 strains exhibited a higher number of aminoglycoside resistance genes compared to non-SC3 strains. Specifically, SC3 strains predominantly carried *aac(3)-XI*, *aph(3'’)-Ib*, *aph(3’)-Ia*, and *aph(6)-Id*, while the prevalence of *aadA1* and *aac(6’)-Ia* was higher in non-SC3 strains (Table S4). Among VGs, significant differences were observed between SC3 and non-SC3 strains (*P* = 0.0059). Specifically, four genes, *spaH*, *spaI*, *srtD*, and *srtE*, exhibited low overall prevalence across all strains but were nearly ubiquitous within the SC3 cluster strains, presenting in 91.07%, 94.64%, 92.86%, and 94.64%, respectively (Figure 2A and Table S4), and can thus be considered SC3-specific virulence genes. Additionally, the prevalence of *fagA*, *fagB*, *fagD*, *spaE*, *spaF*, and *srtC* was significantly higher in the SC3 cluster compared to non-SC3 strains (*P* < 0.05, Table S4).

In terms of isolation time, SC3 strains exhibited a significantly increased isolation rate in 2020-2021, markedly higher than strains isolated in 2017–2018 (15.38% vs. 3.13%, *P* < 0.05). Additionally, among the 44 SC3 strains collected in this study, 14 strains belonged to clone one. To estimate the emergence time of SC3, a potential hypervirulent cluster, we carried out a timed phylogeny reconstruction of the SC3. This analysis suggested that the SC3 strain likely emerged around 1929.56 (95% HPD: 1863.41–1995.71) (Figure 2B and S4).

### Genetic context of four SC3-specific virulence genes

Analyzing the gene environments of *spaH*, *spaI*, *srtD*, and *srtE* revealed that these four genes formed a gene cluster, similarly situated within genomic islands that can be classified into six categories (Figure 2C). In almost all strains (96.30%), the upstream and downstream regions of these four genes contain insertion sequences, IS*3502*, IS*1206*, and IS*1207*, respectively, forming a transposition unit (Figure 2C). Further analysis showed that compared to strain 216 (CP024932), the transposition unit carrying the four genes had integrated into the downstream region of the *lysD* and *asd* genes and the upstream region of the *sigC* and *katA* genes, indicating the potential for these VGs to be horizontally transmitted among *C. striatum* (Figure 2C). Based on this, there were slight differences in the genetic environments of strains 130 and 342, whereas strains 196, 41, and GCA_028527755 exhibited significantly different genetic environments. Notably, Strains 41 and GCA_028527755 lacked the complete set of four genes and insertion sequences (Figure 2C), indicating the variability of these virulence determinants.

### Phenotypic Virulence characterization of SC3-specific virulence genes positive and negative strains

**Figure 3.**
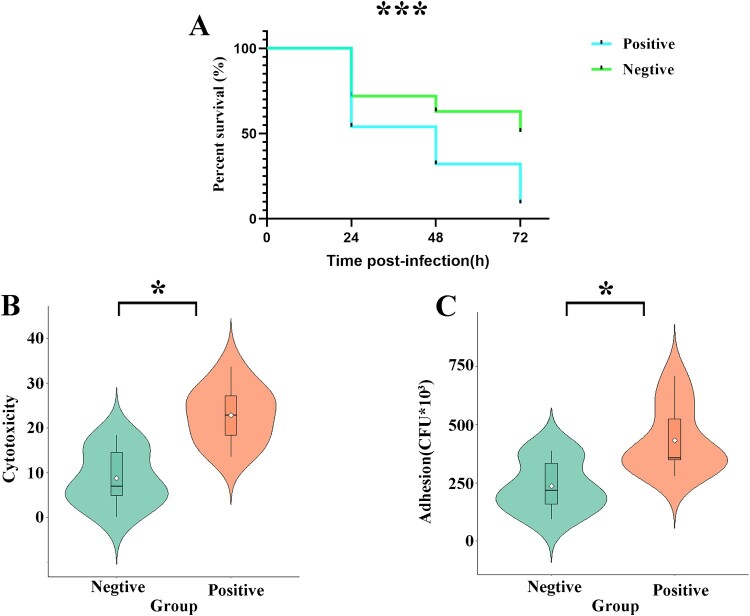
Comprehensive virulence analysis of *C. striatum* strains carrying and not carrying the virulence genes *spaI*, *srtD*, *srtE*, and *spaH*. * represented a *P*-value of <0.05 and *** represented a *P*-value of <0.01. A. The survival curves of G. mellonella after infection of tested *C. striatum* strains. B. The cytotoxicity of tested *C. striatum* strains to A549 cells detected by LDH assay. C. The adherence ability to A549 cells of tested *C. striatum* strains.

To understand the virulence effects of SC3-specific virulence genes on *C. striatum* strains, we conducted virulence experiments on these strains. Based on the phylogenetic tree of the strains and clinical information, we selected 10 strains (8 of which were SC3 strains) carrying the virulence genes *spaI*, *srtD*, *srtE*, and *spaH* as positive strains, and 10 strains lacking these genes as negative strains. The specific strain numbers are listed in Table S3. To verify the virulence phenotypes of these strains, we performed adhesion and virulence assays using both the A549 cell model and the *Galleria mellonella* infection model. The results showed that in the *Galleria mellonella* infection model, the survival rates of larvae injected with positive strains at 24, 48, and 72 hours post-infection were significantly lower than those infected with negative strains (*P* < 0.01, Figure 3A). Similar results were observed in the A549 cell models, where the LDH release assay revealed that positive strains exerted a stronger cytotoxic effect on A549 cells compared to negative strains (*P* < 0.05, Figure 3B). Additionally, a significant reduction in adhesion to A549 cells was observed for the negative strains after 3 hours of incubation, compared to the positive strains (*P* < 0.05, Figure 3C).

### Comparative genomic analysis

**Figure 4.**
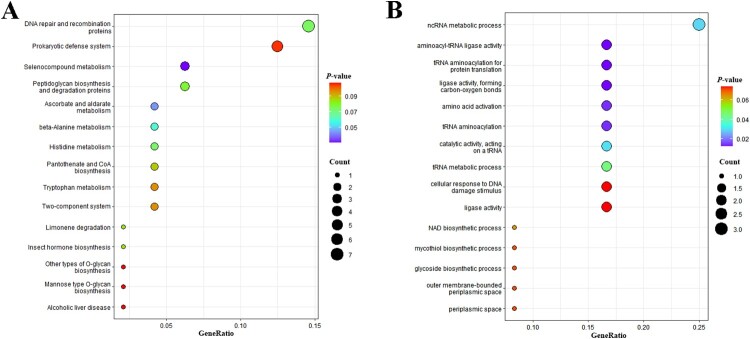
A. KEGG enrichment analysis of unique genes specific to the SC3 strains compared to non-SC3 strains. B. GO enrichment analysis of unique genes specific to the SC3 strains compared to non-SC3 strains.

In all SCs of *C. striatum*, SC3 strains harbored a significantly greater number of CDS, surpassing the cluster average by approximately 160 genes. To investigate whether these additional genes confer an evolutionary advantage to SC3 strains, a comparative genomic analysis was conducted to identify unique genes specific to the SC3 strains compared to all other strains, which were then annotated and subjected to enrichment analysis. The results of the Clusters of Orthologous Groups (COG) annotation indicated a pronounced prevalence of genes in SC3 strains related to replication, recombination, and repair, followed by those involved in amino acid transport and metabolism (Table S6). Additionally, KEGG pathway enrichment analysis indicated these unique genes were mainly enriched in DNA repair and recombination proteins and Prokaryotic defense system pathway, as well as metabolic and biosynthesis (Figure 4A). GO enrichment analysis demonstrated that unique genes predominantly associated with enriched in metabolic and biosynthesis (Figure 4B). To further eliminate the influence of different clades, we also analyzed the unique genes specific to the SC3 strains compared to other sequence cluster strains (SC1, SC17, SC2, SC21, and SC7) within the same clade as SC3 strains. The enrichment analysis results showed similar patterns to the previous findings (Table S7), mainly enriched in DNA repair and recombination proteins, the prokaryotic defense system pathway, and metabolic and biosynthesis processes (Figure S5).

## Discussion

*C. striatum* is a common colonizer in hospital environments especially in ventilators [43]. Recently it has been emerged as an opportunistic pathogen causing nosocomial infections, especially lower respiratory tract infections in both immunocompromised and immunocompetent patients [44]. In this study, we performed an epidemiology and pan-genomic analysis of 263 *C. striatum* isolates and found that some results were worthy attention.

The crude mortality was 13% based on the inpatients data. This was much lower than 40.7% in Korea, 34% in Japan and 52% in China [45–47]. Differences probably lied in the population involved. The Korean study included severe pneumoniae caused by *C. striatum* and the latter two studies collected only *C. striatum* bloodstream infections. On the contrary, we included a wider range sources of *C. striatum*, the patients involved were relatively mild and diverse. Patients suffering from immunocompromised status accounted for 30.6% in our study while in the other three studies, these patients accounted for more than 50%. Consistent with previous investigations, our study showed that *C. striatum* exhibited a high prevalence of MDR phenotypes [18,19,33,48,49]. Almost all strains (98.5%) were classified as MDR. The highest resistance was observed for clindamycin, ciprofloxacin, erythromycin and cephalosporins. Correspondingly, genome sequencing revealed that the most strains carried *erm(X)* which encoded resistance to erythromycin and clindamycin [44]. As for ciprofloxacin, the Ser87Phe and Asp91Ala mutations in the *gyrA* gene were responsible for the resistance of *C. striatum* to quinolones, which is consistent with previous studies [50] The mechanism underlying the high resistance to cephalosporins remains unknown and has not been reported in previous studies, warranting further investigation. Similar to previous studies in China, the US, and Turkey, all strains were susceptible to vancomycin, linezolid and daptomycin [18,19,48]. However, there were also some differences in the resistance rate of individual drugs in different regions. For example, high resistance against erythromycin ranging from 79% in Turkey to 93.5% in our study while all 67 *C. striatum* strains were susceptible to erythromycin in Korea [17,48]. Meanwhile, the MDR rate in the Korean study was also relatively low, with 77.6% isolates showing MDR phenotype [17]. Possible explanations may be the variations in geographic positions and the source of strains. Notably, most strains were isolated from sputum both in our study and in studies from the US and Turkey [19,48]. In the Korean study, the most common clinical specimens were urine (35.8%) and skin abscesses (32.8%) [17]. The abundance of mobile genetic elements and integrative conjugative elements distributed in the genomes suggested *C. striatum* isolates might evolve to MDR phenotypes through horizontal gene transfers.

Nosocomial outbreak of *C. striatum* infections has been reported in various countries including Brazil, Italy, Belgium and China [12,14,16,18,33,51]. Multiple tools such as MALDI-TOF MS, repetitive-sequence-based PCR, pulsed-field gel electrophoresis (PFGE) and more recently WGS, have been applied to unveil the genetic relatedness between strains. Before the easy access of WGS, PFGE was the most common tool for tracking nosocomial outbreak of *C. striatum* infections [12,14,51]. In this study, we conducted a pan-genome analysis of *C. striatum* isolates and defined literally for the first time the clonal transmission standard for *C. striatum* as groups with fewer than 20 SNPs and consisting of more than 10 strains. The SNP-based phylogenetic analysis revealed that multiple clones contributed to nosocomial transmission of *C. striatum*. In contrast to the previous study which showed that within-unit transmission was more common than between-unit transmission in three regions of China including Beijing [18], our study displayed a higher inter-ward transmission than intra-ward. A possible difference may lie in the source of strains. Strains in the previous study were isolated during 2016-2018, the pre-COVID-19 era, while strains in our study were isolated during 2021-2022, the post-COVID-19 era. It is known that the COVID-19 contributed much to the patient mobility between wards as all wards were open to COVID-19 patients in that circumstance, which possibly contributing to inter-ward transmission events. In addition, more than 50% of the isolates we isolated from patients in the emergency department and intensive care unit, in which the patients were highly mobile. For example, if the patients’ circumstances became not so critical but not qualified for discharge, they would be assigned to a department most related to his primary disease, thus contributing to the transmission occurred between patients, between health workers, through fomites or the environment. The ubiquity of different clones appearing in various departments during the whole study period suggested that *C. striatum* had persisted in the hospital environment for a long time. Similar to previous studies, the most at-risk units for intra- and inter-unit transmission were emergency, intensive care unit, respiratory, neurosurgery and neurology [33,48]. These findings underscore the importance of effective control measurements such as sputum culture surveillance of *C. striatum* within these units.

Bayesian analysis of strains from this study and those available in NCBI database further identified SC3, a potentially hypervirulent and MDR cluster of *C. striatum*. The hypervirulence of SC3 was suggested based on the high prevalence and even uniqueness of multiple VGs distributed in SC3. Gene annotation revealed that these genes were mainly related to DNA repair and recombination, suggesting an essential role in bacteria metabolic and biosynthesis. Noticeably, four genes *spaH*, *spaI*, *srtD* and *srtE* were suggested as SC3-specific virulence genes. Among these, *spaH* and *spaI* were reported to be involved in the synthesis of pili; *srtD* and *srtE* encoding sortases were devoted to the assembly of pili in *C. diphtheriae* [52,53]. Pili play an important role in bacteria adhesion, which is the first and essential step in biofilm formation and host infection [54,55]. Previous studies have demonstrated that *C. striatum* has the ability to form biofilms and exhibited good adhesion to human epithelial cells [15,56]. Therefore, it can be anticipated that our virulence experiments showed that these VGs in SC3 may contribute to its virulence and pathogenicity. Moreover, our genetic context analysis revealed for the first time that the four genes formed a transposition unit along with insertion sequences, indicating the role of horizontal and vertical gene transfer of virulent strains. It should be alarmed since *C. striatum* were highly MDR, the mobility of VGs in additions to ARGs added to the transmission risk of such strains, leading to intractable clinical situations. A significantly increased isolation rate of SC3 was also observed with timeline, and 31.82% of SC3 strains in this study belong to the same clone one, suggesting a potential evolutionary advantage and enhanced transmission capability within the SC3 strains. We also reviewed the clinical characteristics of the 44 patients isolating SC3 strains in this study and found that one patient developed disseminated *C. striatum* bloodstream infection leading to final death. However, since most of the strains isolated were considered as colonization, the actual pathogenic role of these strains were worth further in vitro and in vivo experiments. Current findings of SC3 may shed light on the virulence of *C. striatum* that were scarcely explored, offering a valuable tool to select for alternative therapeutic targets.

There were some limitations of this study. First, despite the relatively large number of strains, it was still a single center study that may lack representativeness. Second, most strains were isolated from sputum and considered as colonization, there were not enough data for the clinical and genetic characteristics of strains causing infections. More strains from multiple centers and diverse samples were expected to provide more sufficient data in the future.

## Supplementary Material

Supplementary Figures.docx

Supplementary Tables 0807 cjw zml.xlsx

## Data Availability

All assembled sequence data have been deposited in GenBank under the BioProject accession number PRJNA1212338 and Genome Warehose in National Genomics Data Center, Beijing Institute of Genomics, Chinese Academy of Sciences China/National Center for Bioinformation, under the BioProject accession number PRJCA040702.
