# Supplementary material for: Whole genome sequencing reveals significant intra-hospital clonal transmission and a potential multidrug resistant and hypervirulent sequence cluster of Corynebacterium striatum
Source: Emerg Microbes Infect. 2025 Oct 14;14(1):2563795. doi: 10.1080/22221751.2025.2563795 (PMC12529752; doi:10.1080/22221751.2025.2563795)
Supplement: Supplementary Figures.docx [file TEMI_A_2563795_SM6279.docx]

Supplementary Materials for

**Whole Genome Sequencing Reveals Significant Intra-Hospital Clonal Transmission and a Potential Multidrug Resistant and Hypervirulent Sequence Cluster of *Corynebacterium Striatum***

Menglan Zhou, Jiawei Chen *et al.*

*Corresponding author. Email: [sunhl2010@sina.com,](mailto:liuyluijk@163.com,) zhengyinl@hotmail.com.

**This file includes:**

Supplementary Methods and Supplementary Figs. S1 to S4.


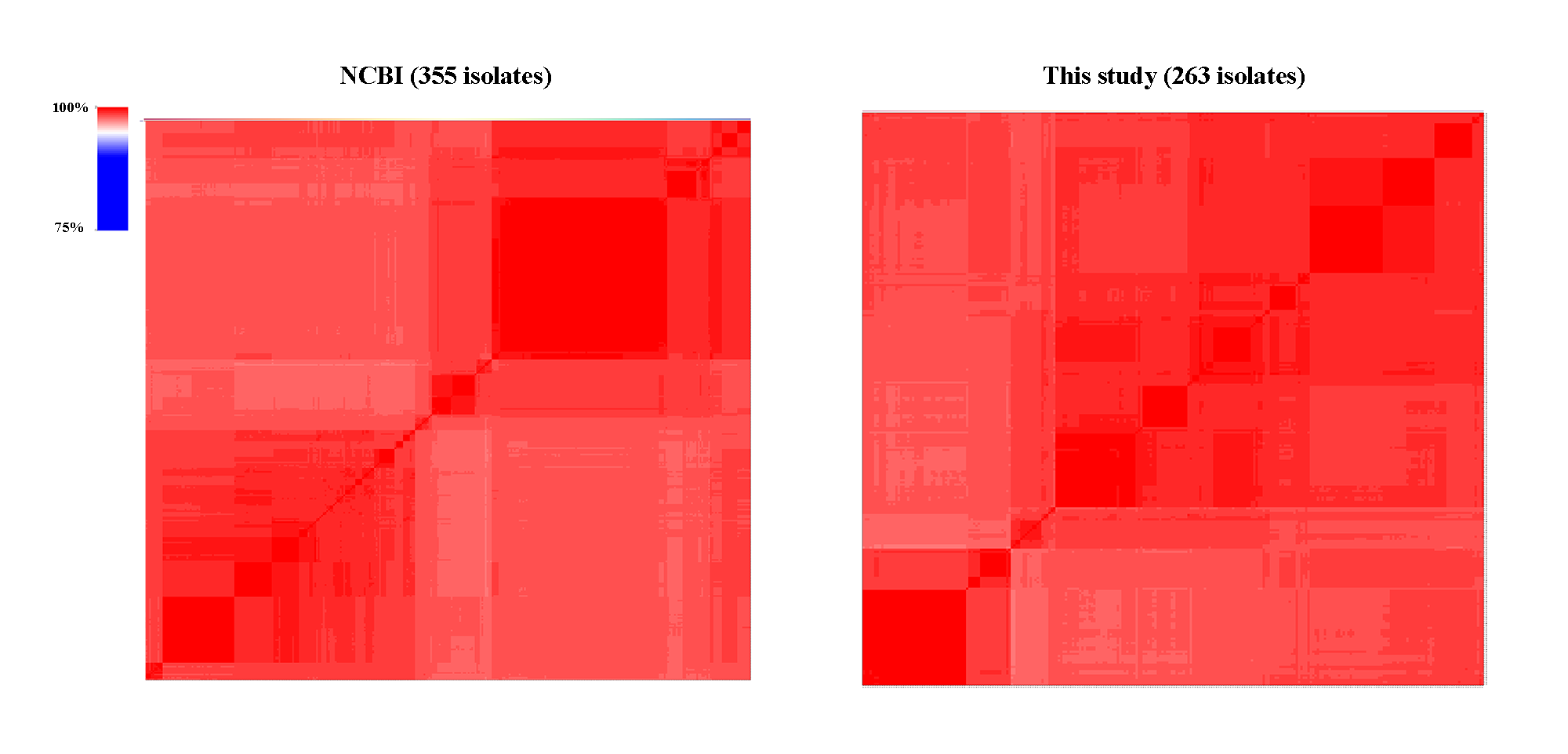


**Figure S1.** Pairwise average nucleotide (ANI) identity comparison was calculated for all *Corynebacterium Striatum* isolates shown on a heatmap with blue indicating low and red indicating high nucleotide identity. Strains on the left are sourced from NCBI, while those on the right are from this study.


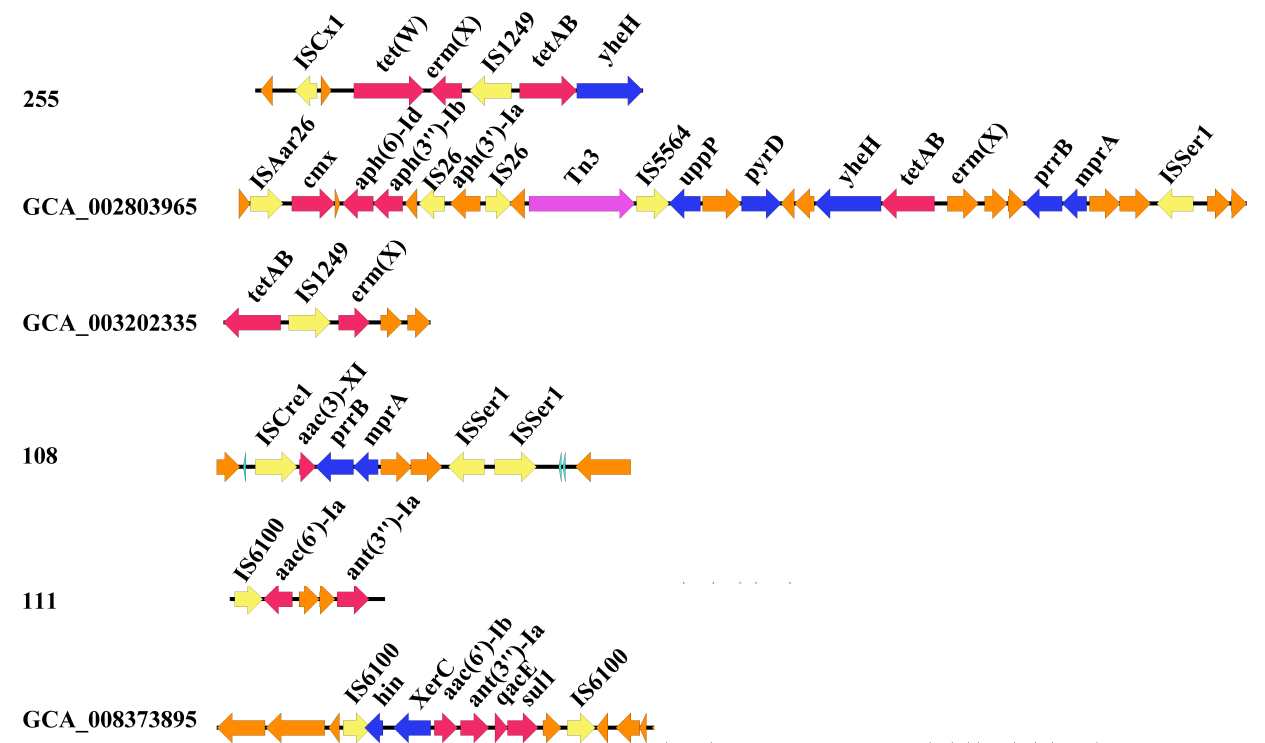


**Figure S2.** The representative genomic context of macrolide, lincosamide, tetracycline, and aminoglycoside resistance genes, along with their associated mobile genetic elements. The red arrows represent the resistance genes, the yellow arrows indicate insertion sequences, and the purple arrows denote transposons. The blue arrows point to functional genes, while the grey arrows represent hypothetical proteins.


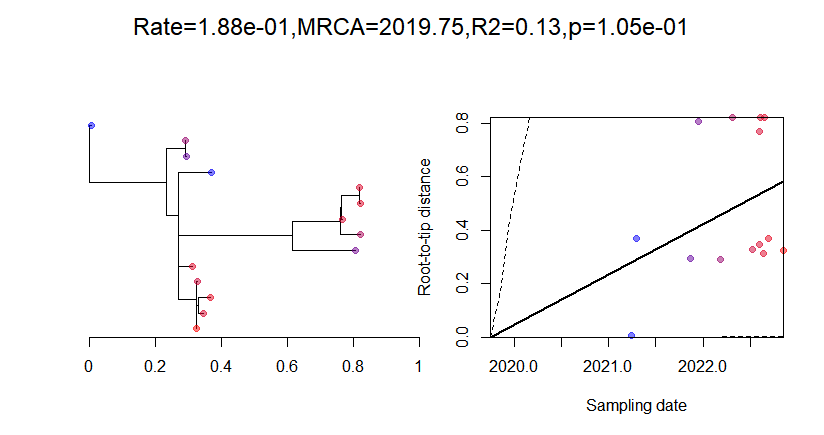


**Figure S3.** Regression of root-to-tip distance against sampling time shows temporal signal in clone one strains isolated from this study. *P*-value indicated the statistical significance after 10,000 date randomizations.


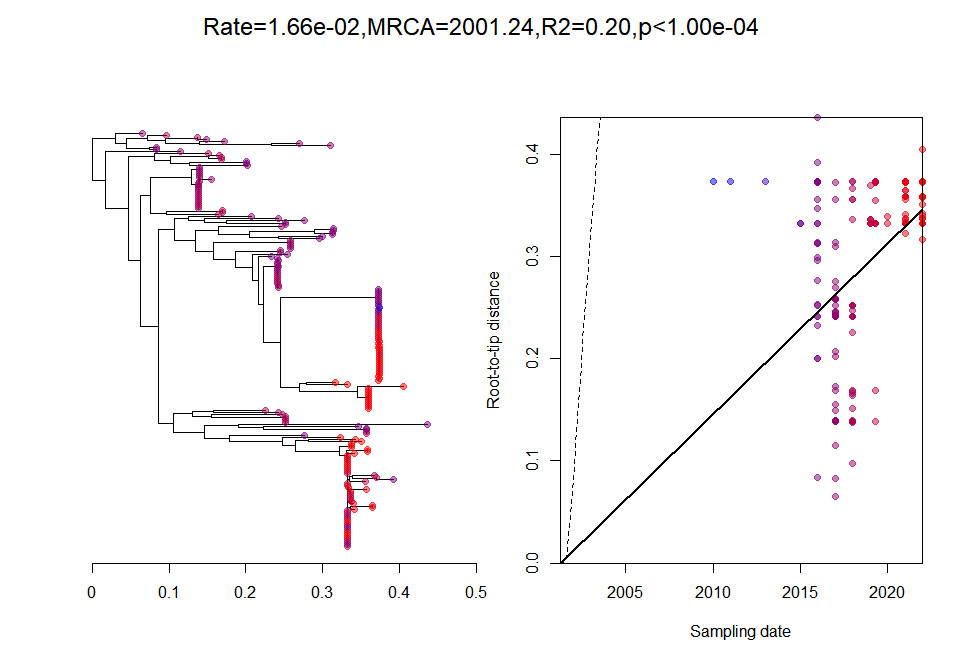


**Figure S4.** Regression of root-to-tip distance against sampling time shows temporal signal in SC3 strains and other sequence cluster strains (SC1, SC17, SC2, SC21, and SC7) from the same clade as SC3 strains. *P*-value indicated the statistical significance after 10,000 date randomizations.


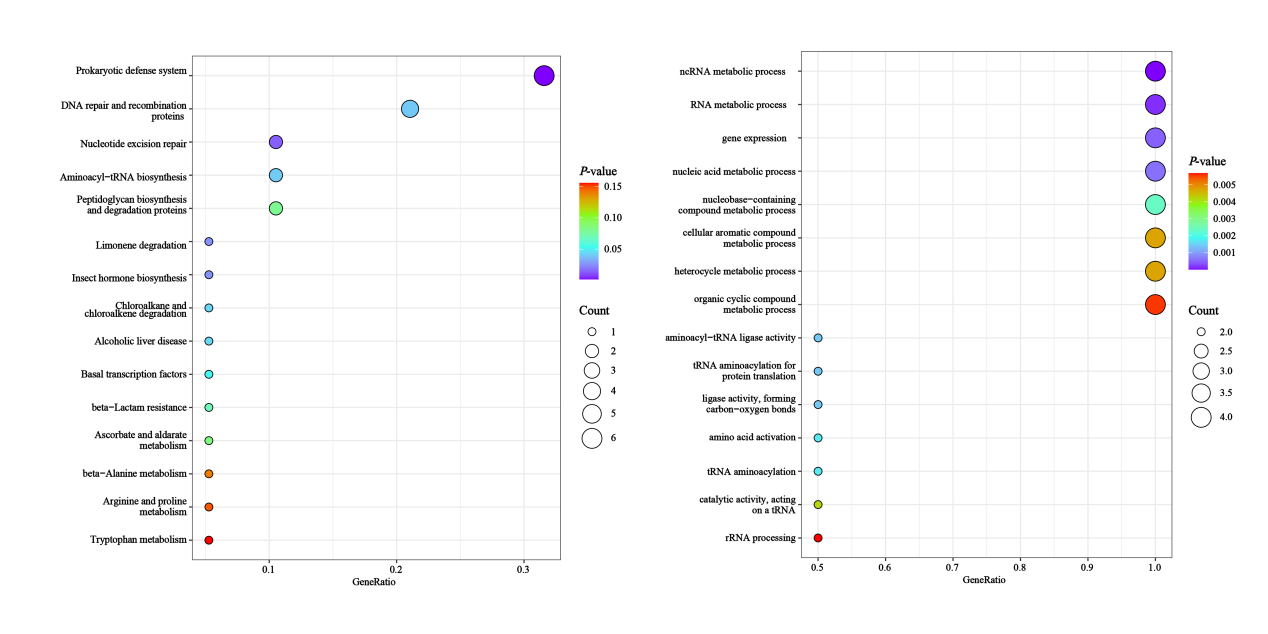


**Figure S5. A.** KEGG enrichment analysis of unique genes specific to the SC3 strains, compared to other sequence cluster strains (SC1, SC17, SC2, SC21, and SC7) within the same clade. **B.** GO enrichment analysis of unique genes specific to the SC3 strains compared to non-SC3 strains.
